# Pharmacotherapics Advice in Guidelines for COVID-19

**DOI:** 10.3389/fphar.2020.00950

**Published:** 2020-06-24

**Authors:** Zhang-Ren Chen, Ying Zhou, Jin Liu, Hong-Wei Peng, Jian Zhou, Hai-Li Zhong, Li-Li Liu, Ming-Fang Lai, Xiao-Hua Wei, Jin-Hua Wen

**Affiliations:** Department of Pharmacy, The First Affiliated Hospital of Nanchang University, Nanchang, China

**Keywords:** COVID-19, pneumonia, guideline, pharmacotherapy, SARS-CoV-2

## Abstract

Since December 2019 to May 2020, coronavirus disease 2019 (COVID-19) has infected over 6 million people worldwide. Due to its sudden and rapid outbreak, effective treatment for COVID-19 is scarce. Based on national clinical trials of novel treatments, China, Italy, Germany, and other countries and organizations have published multiple guidelines for COVID-19 and advised many medicines, such as chloroquine and tocilizumab. In this paper, we summarize the pharmacotherapy for COVID-19 according to those guidelines, highlight updates of the pharmacotherapy guidelines, and review the efficacy and safety of the indicated anti-COVID-19 drugs.

## Introduction

SARS-CoV-2 (previously termed 2019 novel coronavirus, 2019-nCoV), a virus that causes COVID-19, likely initially transmitted from bat to human (Gorbalenya et al., 2020), infected over 6 million people worldwide from its outbreak in December 2019 to May 2020 (China CDC, 2020; WHO, 2020a). Due to the relatively high basic reproduction number (R_0_) of the 2019-nCoV virus (Liu et al., 2020), COVID-19 spread rapidly and has already become a pandemic. The World Health Organization (WHO) has declared a Public Health Emergency of International Concern (PHEIC) for COVID-19 (WHO, 2020b) and has announced that COVID-19 is a pandemic (National Public Radio, 2020). Controlling the transmission and treating the infected cases is of great urgency.

**Figure 1:**
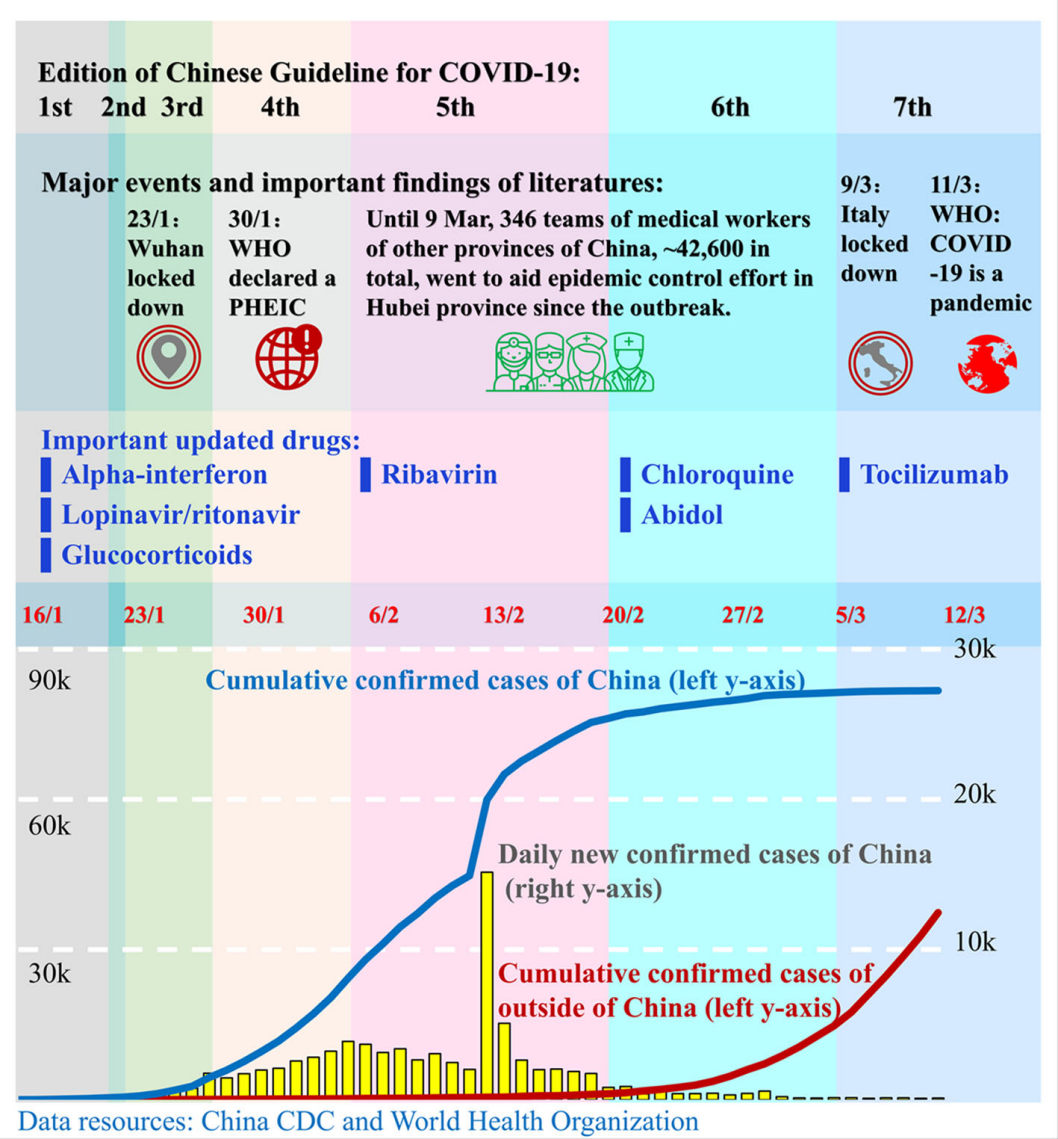
Timeline of the publication of the Chinese Guidelines for COVID-19. Since the outbreak of COVID-19 in December 2019, the governments of China and other countries and the World Health Organization (WHO) have taken actions to stop the transmission of the SARS-CoV-2 virus and to treat the infected patients. China's government published seven editions of guidelines for the diagnosis and treatment of COVID-19 from 16 February to 4 March. Those guidelines advised a number of novel drugs for the treatment of COVID-19. Red numbers in the middle are dates. The blue line and red line represent the cumulative confirmed COVID-19 cases in China and outside of China, respectively, and correspond to the left y-axis. The yellow bars represent the daily new confirmed cases in China and correspond to the right y-axis. Each colored column presents a corresponding edition of the Chinese Guidelines. The position of drugs in the 3rd row corresponds to the edition in which the drugs were recommended for the first time.

To develop treatments for COVID-19, many marketed drugs have been re-purposed for usage in the clinic. China, Italy, Germany, the ATS (American Thoracic Society), the SSC (Surviving Sepsis Campaign), the NIH (National Institutes of Health), the IDSA (Infectious Diseases Society of America), and the FDA (Food and Drug Administration) released guidelines and recommended several medicines for the treatment of COVID-19 (**Table 1**). Since January 16, 2020, China's government has published seven editions of guidelines for the diagnosis and treatment of 2019 novel coronavirus (2019-nCoV)-infected pneumonia (hereinafter referred to as the Chinese Guidelines, **Table 2**, **Figure 1**) (The national commission of the people's republic of China, 2020a; The national commission of the people's republic of China, 2020b; The national commission of the people's republic of China, 2020c; The national commission of the people's republic of China, 2020d; The national commission of the people's republic of China, 2020e; The national commission of the people's republic of China, 2020f; The national commission of the people's republic of China, 2020g) and two editions of guidelines for the diagnosis and treatment of serious and critical 2019 novel coronavirus (2019-nCoV)-infected pneumonia (The national commission of the people's republic of China, 2020h; The national commission of the people's republic of China, 2020i). To date, there is still no approved specific antiviral drug for COVID-19, and providing supportive care to help relieve symptoms is the most vital strategy for COVID-19 treatment. In this article, we summarize the pharmacotherapy guidelines for COVID-19, highlight the updates to the pharmacotherapy guidelines, and reviewed the efficacy and safety of the anti-COVID-19 drugs recommended.

**Table 1:** The pharmacotherapies advised by official guidelines for COVID-19.

Country/Organization	IFN	Ribavirin	Arbidol	Anti-HIV drugs	HCQ/CQ	Azithromycin	Remdesivir	Corticosteroids	Tocilizumab	Other Drugs	Update time	References
China (7th edition)	Yes	Yes	Yes	Yes(L/r)	Yes			Severe and critical cases	Yes	*(Detailed in* **Table 2***)*	2020.03.04	(The National Commission of the People's Republic of China, 2020i)
German				Compassionate use (L/r)	Compassionate use		Compassionate use			Camostat (compassionate use)	2020.04.14	(Kluge et al., 2020)
Italy 2.0				Yes (L/r, Darunavir, Darunavir/cobicistat)	Yes		Yes	Only patients with ARDS	Yes		2020.03.13	(Italian Society of Infectious and Tropical Diseases SECTION, 2020)
ATS				No suggestion	Conditional (on a case-by-case basis)		No suggestion	No suggestion	No suggestion		2020.04.03	(Wilson et al., 2020)
NIH	Only in clinical trial			Only in clinical trial	Only in clinical trial	Only in clinical trial	For severe cases; Not for mild or moderate cases	Only patients with refractory shock		Janus kinase inhibitors (e.g., baricitinib, in clinical trial)	2020.05.12	(NIH, 2020)
IDSA	Only in clinical trial	Only in clinical trial	Only in clinical trial	Only in clinical trial	Only in clinical trial	Only in clinical trial	Only in clinical trial	Only patients with ARDS	Only in clinical trial		2020.04.21	(Bhimraj et al., 2020)
FDA					EUA (both HQ & CQ)		EUA				2020.05.16	(FDA, 2020)
SSC								Only patients with ARDS		Antimicrobials/antibacterial agents, acetaminophen/paracetamol	2020.03.28	(Alhazzani et al., 2020)

Key: ATS, American Thoracic Society; SSC, Surviving Sepsis Campaign; NIH, National Institutes of Health; IDSA, Infectious Diseases Society of America; FDA, Food and Drug Administration; ARDS, Acute Respiratory Distress Syndrome; IFN, interferon; EUA, emergency use authorization; HCQ, hydroxychloroquine; CQ, chloroquine; L/r, Lopinavir/ritonavir.

**Table 2:** Updates of the pharmacotherapy recommendations of the Chinese Guidelines for COVID-19 (1st to 7th edition).

Edition	Updated Date	General treatments				Treatments for Severe and Critical cases	Major Changes
		Antiviral Drugs	Antibacterial Drugs	Glucocorticoids	TCM		
1st	2020/1/16	**Alpha-interferon**: 5 million U inhalation bid; **Lopinavir/ritonavir**: Lopinavir 400 mg-ritonavir 100 mg orally bid.	Avoid blind or inappropriate use of antimicrobials, especially the combination of broad-spectrum antimicrobials	A short period of time (3 to 5 days) as appropriate. <1 to 2 mg/kg/d of methylprednisolone	Dialectical treat according to the syndromes of patients	–	–
2nd	2020/1/21	*(Same as the previous edition)*	*(Same as the previous edition)*	*(Same as the previous edition)*	*(Same as the previous edition)*	–	–
3rd	2020/1/22	*(Same as the previous edition)*	*(Same as the previous edition)*	*(Same as the previous edition)*	–	–	TCM was listed in a single part
4th	2020/1/27	*(Same as the previous edition)*	*(Same as the previous edition)*	–	–	**Glucocorticoids**: dose for a short period of time (3 to 5 days); <1-2 ml/kg/day methylprednisolone; **Xuebijing**: 100 mL/d iv injection, tid; **Intestinal microecological regulators**; **Convalescent plasma therapy**	Glucocorticoids were removed from general treatments and recommended for serious and critical cases only
5th	2020/2/8	**Alpha-interferon**: 5 million U inhalation tid; **Lopinavir/ritonavir**: Lopinavir 400 mg-ritonavir 100 mg orally tid. **Ribavirin**: 500mg, bid/tid ivgtt.	*(Same as the previous edition)*	–	–	*(Same as the previous edition)*	Ribavirin and convalescent plasma therapy
6th	2020/2/19	**Alpha-interferon**: 5 million U inhalation tid; **Lopinavir/ritonavir**: Lopinavir 400 mg-ritonavir 100 mg orally tid. **Ribavirin**: 500 mg, bid/tid ivgtt. In combination with interferon and lopinavir/ritonavir; ≤10 days; **Chloroquine:** 500 mg orally bid, ≤10 days; **Abidol**: 200 mg orally tid	*(Same as the previous edition)*	–	–	*(Same as the previous edition)*	Chloroquine phosphate and abidol
7th	2020/3/4	*(Same as the previous edition)*	*(Same as the previous edition)*	–	–	**Glucocorticoids**; **Xuebijing**; **Intestinal microecological regulators**; **Convalescent plasma therapy**; **Tocilizumab:** 4-8 mg/kg first dose, 400 mg recommended; if the first medication is not effective, dose once more 12 hours later (dose as before); maximum: 2 times, and 800 mg per day **Intravenous immunoglobulin gamma (IVIg):** ivgtt for children with severe and critical cases	Tocilizumab (immunotherapy)

## Antiviral Drugs

The majority of anti-SARS-CoV-2 virus drugs are adopted from the treatment of severe acute respiratory syndrome (SARS) or Middle East respiratory syndrome (MERS): alpha-interferon, lopinavir/ritonavir, and ribavirin. Besides, novel antiviral drugs have also been applied in the clinic, such as chloroquine. Considering the safety and toxicity of those antiviral drugs, the combination of three or more antiviral drugs is not recommended. Most antiviral treatments still lack high-level evidence to support antiviral efficacy. Clinicians should balance the benefits and risks of such drugs before medication.

### Remdesivir

Remdesivir, a nucleoside analog, inhibits RNA replicase polyprotein 1ab (Agostini et al., 2018). It was originally developed for the treatment of the Ebola virus (Warren et al., 2016). Remdesivir has a superior anti-MERS activity compared to lopinavir and ritonavir *in vitro* and *in vivo* (Sheahan et al., 2020) and displayed anti-SARS-CoV-2 ability *in vitro* (Wang M. et al., 2020). The first COVID-19 case in the USA used remdesivir, and clinical condition improved from the 2nd day of remdesivir medication (Holshue et al., 2020). It has been assessed in RCTs in both China and the USA (NIH, 2020). However, it showed different results in China and the USA. Remdesivir was not associated with statistically significant clinical benefits or adverse events for 237 adult patients admitted to hospital for severe COVID-19 in the RCT enrolled in China (Wang Y. et al., 2020). In the USA, compassionate use of remdesivir displayed clinical improvement in 36 of 53 hospitalized severe COVID-19 patients (Grein et al., 2020) and 14 of 17 patients in the infectious disease ward (Antinori et al., 2020). In an RCT with 1063 patients enrolled in the USA, remdesivir was superior to placebo in shortening the time to recovery and evidence of lower respiratory tract infection; also, an ethnic difference was observed: remdesivir is effective among white patients and is not significantly effective among black and Asian patients (Beigel et al., 2020). The guideline from Italy recommended remdesivir (Italian Society of Infectious and Tropical Diseases SECTION, 2020), and the FDA approved emergency use authorization (EUA) of remdesivir for the treatment of COVID-19 (FDA, 2020). In short, remdesivir is helpful but not a wonder drug for COVID-19 patients (Mahase, 2020).

### Favipiravir

Favipiravir is a modified pyrazine analog and was initially approved for therapeutic use in resistant cases of influenza. It targets RNA-dependent RNA polymerase (RdRp) enzymes and inhibits the transcription and replication of viral genomes (Furuta et al., 2017). Cai et al. found that favipiravir showed significant improvement in chest imaging compared with lopinavir/ritonavir for the treatment of COVID-19 (35 vs. 45 patients). Chen et al. found the favipiravir did not significantly improve the clinical recovery rate at Day 7 compared to arbidol but significantly improved the latency to relief for pyrexia and cough in a 120 vs. 120 patient trial (Chen et al., 2020). However, Lou et al. did not observe clinical improvement with favipiravir treatment for COVID-19 patients (Lou et al., 2020). There is still a lack of sufficient evidence to support the clinical anti-COVID-19 effects of favipiravir.

### Chloroquine and Hydroxychloroquine

Chloroquine and hydroxychloroquine are canonical quinoline antiparasitic drugs, indicated to treat infections of malaria and also used off-label for the treatment of rheumatic diseases and lupus erythematosus. Besides, Andrea Savarino et al. demonstrated the potential therapeutic benefits of chloroquine in viral diseases (Te et al., 2007), including SARS (Savarino et al., 2003). Thus, it has been re-purposed for the prophylaxis and treatment of Zika virus infection (Li et al., 2017). Wang et al. reported that chloroquine effectively inhibits SARS-CoV-2 infection by increasing endosomal pH and interfering with the glycosylation of cellular ACE2 receptors (Wang M. et al., 2020). The blood concentration of chloroquine can reach the EC_90_ value of anti-SARS-CoV-2 with regular dosing for rheumatoid arthritis (Wang M. et al., 2020). In several clinical trials, chloroquine has been proved to inhibit the SARS-CoV-2 virus *in vivo* (Gao et al., 2020). Therefore, it was included in the Chinese Guidelines for the first time in the 6th edition. Concerning the toxicity of chloroquine, the dose recommended by the Chinese Guidelines was updated in the 7th edition (Riou et al., 1988). Also, an expert consensus statement from Shanghai recommended hydroxychloroquine instead of chloroquine (Cutler et al., 1988; Shanghai Clinical Treatment Expert Group for corona virus disease 2019, 2020). The Italian guidelines recommended chloroquine and hydroxychloroquine (when chloroquine is not available) (Italian Society of Infectious and Tropical Diseases SECTION, 2020). The FDA approved the EUA of chloroquine and hydroxychloroquine for the treatment of COVID-19 (FDA, 2020). However, there is still controversy about the effect and safety of chloroquine and hydroxychloroquine. Mehra and colleagues have not found a benefit of hydroxychloroquine or chloroquine, when used alone or with a macrolide, for in-hospital outcomes for COVID-19(N=96 032) (Mehra et al., 2020). In an observational study with 1446 hospitalized patients, hydroxychloroquine administration was not associated with either a greatly lowered or an increased risk of the composite endpoint of intubation or death (Tang et al., 2020). The American College of Physicians suggested that clinicians should not use chloroquine or hydroxychloroquine alone or in combination with azithromycin as a treatment of patients with COVID-19 due to the known harms and there being no available evidence of benefits in patients with COVID-19 (Qaseem et al., 2020).

### Abidol

Abidol, also called umifenovir, is a broad-spectrum antiviral drug developed by a Russian pharmaceutical company and approved for marketing in Russia and China (Brooks et al., 2012). It was licensed for the prophylaxis and treatment of influenza and other respiratory viral infections (Brooks et al., 2012). Its mechanisms of antiviral action including interactions with certain amino acid residues to form a hydrophobic aromatic stacking structure and interactions with aromatic residues of the viral glycoproteins involved in fusion and cellular recognition(Kadam and Wilson, 2017; Zeng et al., 2017). Its antiviral ability in the treatment of various virus infections, including SARS (Khamitov et al., 2008) and Ebola virus (Hulseberg et al., 2019), has been studied and proved. Some studies have observed anti-COVID-19 potential *in vitro* and in clinic (Wang Z. et al., 2020). Chinese Guidelines recommended abidol for anti-COVID-19 treatment from the 6th edition. A clinical trial from Shanghai did not find any effects of lopinavir/ritonavir and abidol in terms of relieving symptoms or accelerating viral clearance (Jun et al., 2020). A retrospective study showed that abidol might not improve the prognosis or accelerate SARS-CoV-2 clearance in non-ICU patients (Lian et al., 2020).

### Anti-Human Immunodeficiency Virus (HIV) Drugs (Lopinavir/Ritonavir and Darunavir/Cobicistat)

Anti-HIV combination antiviral drugs, mainly lopinavir/ritonavir and darunavir/cobicistat, have been re-purposed as anti-SARS-CoV-2 agents. Both lopinavir and darunavir are inhibitors of the 3CLpro protease of coronavirus, a key protein that cleaves the large replicase polyproteins during viral replication. Ritonavir increases the bioavailability of lopinavir by cytochrome P450 isoenzyme 3A4 (CYP3A4) (Mangum and Graham, 2001), and cobicistat is a more selective cytochrome P450 3A inhibitor than ritonavir without enzyme-inducing properties (Kakuda et al., 2015). Lopinavir/ritonavir has shown anti-coronavirus activity *in vitro*, in animal model, and in non-randomized trials (Chan et al., 2003; Chu et al., 2004; Chan et al., 2015; Zumla et al., 2016). For COVID-19, lopinavir/ritonavir has been recommended by Chinese Guidelines since the first edition and is also recommended in the guidelines of Italy; the German, ATS, NIH, SSC, and IDSA guidelines do not recommend it (**Table 1**). It has also been applied for anti-COVID-19 treatment in South Korea (Lim et al., 2020) and Thailand. However, there is still controversy about its efficacy and safety (Jun et al., 2020; Kim, 2020). Cao et al. did not observe any benefit with lopinavir-ritonavir treatment in 199 hospitalized adult patients with severe COVID-19 (Cao et al., 2020), and Cheng et al. found that lopinavir/ritonavir did not shorten the duration of SARS CoV-2 shedding (Cheng et al., 2020). Lopinavir/ritonavir can induce liver damage when treating COVID-19 (Fan et al., 2020). Considering the toxicity of lopinavir/ritonavir, Chinese Guidelines have warned that its adverse reactions should be considered carefully since the 5th edition and have stated that the treatment should last less than 10 days since the 6th edition; the Italian guidelines recommend both lopinavir/ritonavir and darunavir/cobicistat (Italian Society of Infectious and Tropical Diseases SECTION, 2020). However, according to the case report of Riva et al., darunavir does not prevent SARS-CoV-2 infection in HIV patients (Riva et al., 2020). The triple combination of interferon-β-1b, lopinavir/ritonavir, and ribavirin was found to be safe and superior to lopinavir/ritonavir alone in alleviating symptoms and shortening the duration of viral shedding and hospital stay in patients with mild to moderate COVID-19 (Hung et al., 2020).

### Interferons (IFN)

Interferons are broad-spectrum antiviral drugs that regulate the immune system and inhibit the replication of the virus. IFN-α has been applied for the treatment of coronavirus diseases previously, such as for the treatment of SARS (Haagmans et al., 2004) and MERS (Al Ghamdi et al., 2016). IFN-α has been recommended as an anti-SARS-CoV-2 virus drug in the Chinese Guidelines since the first edition. Besides, the expert consensus statement from Shanghai recommended IFN-κ rather than IFN-α (Shanghai Clinical Treatment Expert Group for Coronavirus Disease 2019, 2020). A Hong Kong group used IFN-β1b (Hung et al., 2020), and some groups suggested that IFN-λ might be helpful for anti-SARS-CoV-2 treatment (Andreakos and Tsiodras, 2020; Prokunina-Olsson et al., 2020).

### Ribavirin

Ribavirin is a prodrug and guanosine analog. It was metabolized into active metabolites, the deribosylated base, and its 5'-phosphate derivatives, by host cell enzymes after absorption (Wu et al., 2003). The mechanisms of the antiviral effect of ribavirin including 1) inhibiting the shift from Th2 to Th1 immune response, 2) inhibiting host inosine monophosphate dehydrogenase (IMPDH) and causing a decrease in GTP, 3) directly inhibiting the replication of the virus, and 4) inhibiting the mutagenesis of virus (Te et al., 2007). Its triple combination with interferon-β-1b and lopinavir/ritonavir might be more helpful for anti-COVID-19 treatment compared to lopinavir/ritonavir alone (Hung et al., 2020).

### Transmembrane Protease, Serine 2 (TMPRSS2) Inhibitors

The host cell entry of SARS-CoV-2 depends on the ACE2 receptor and protease TMPRSS2. Hoffmann et al. demonstrated that TMPRSS2 inhibitors may block the entry of the virus and that a marketed drug, camostat, might be a potential antiviral treatment for COVID-19 (Hoffmann et al., 2020). German guidelines mentioned the compassionate use of camostat (Kluge et al., 2020).

## Antibacterial Drugs

Both Chinese and German guidelines recommended that blind or inappropriate use of antibacterial drugs, especially the combination of broad-spectrum antibacterial drugs, should be avoided in the clinic (The national commission of the people's republic of China, 2020g). Enhancement of bacteriological surveillance should be performed, and appropriate antibacterial drugs should be given promptly when secondary bacterial infection occurs (Jin et al., 2020).

## Glucocorticoids

Glucocorticoids are commonly used for the treatment of SARS owing to their anti-inflammatory and immunosuppressive effects (Loutfy et al., 2003). Since glucocorticoids induce severe sequelae, such as osteonecrosis of the femoral head (Griffith et al., 2005) and mental disorder (Hong et al., 2009), and decelerate the clearance of virus, glucocorticoids were removed from the Chinese Guidelines and not recommended for treatment of mild COVID-19 patients from the 4th edition onwards. For serious and critical patients with progressive deterioration of the oxygenation index, rapid progression as shown in imaging, and excessive activation of the body's inflammatory response or patients with acute respiratory distress syndrome (ARDS), short-term use of glucocorticoids is recommended by the Chinese, Italian, IDSA, and SSC guidelines.

## Tocilizumab

Tocilizumab is a recombinant, humanized, anti-IL-6 receptor monoclonal antagonist (antibody). It is approved for the treatment of cytokine release syndrome (CRS), active rheumatoid arthritis (RA), giant cell arteritis (GCA), active polyarticular juvenile idiopathic arthritis (PJIA), and active systemic juvenile idiopathic arthritis (SJIA) (FDA) (FDA ACTEMRA, 2020). It has a significant therapeutic effect on life-threatening cytokine release (Le et al., 2018). The 7th edition of the Chinese Guidelines recommended tocilizumab as an immunotherapy for patients with extensive lung lesions or severely ill patients who have elevated IL-6 levels. Allergic reactions should be considered. Xu et al. observed that tocilizumab improved the clinical outcome immediately in severe and critical COVID-19 patients and that it was an effective treatment to reduce mortality (Xu X. et al., 2020). A systematic review showed benefits from tocilizumab to COVID-19 (Alzghari and Acuña, 2020).

## Immunomodulatory Drugs and Intestinal Microecological Preparations

Due to the immune deficiency of some severe patients, it is necessary to strengthen their immune capability. The 2nd edition of the Chinese Guidelines recommended Thymosin alpha-1 as an immunomodulatory drug (Du et al., 2018) for severe patients with low lymphocyte counts and serious and critical cases with low cellular immunity (The national commission of the people's republic of China, 2020i). Intravenous immunoglobulin gamma (IVIg), a plasma-made mixture of IgG1 and other antibodies (Baerenwaldt et al., 2010), was recommended for severely ill patients, especially children (The national commission of the people's republic of China, 2020i). Intestinal microecological regulators were recommended for maintaining the intestinal microecological balance and preventing secondary bacterial infections (Hidalgo-Cantabrana et al., 2017; The national commission of the people's republic of China, 2020g; The national commission of the people's republic of China, 2020h).

## Traditional Chinese Medicines (TCM)

The Chinese Guidelines have recommended a set of TCM in a single section since the 3rd edition. Those recommended drugs include Jinhua Qinggan Granules, Lianhua Qingwen Capsule (Mingxing Huang et al., 2020), Shufeng Jiedu Capsule, and other mentioned herbal drugs (Jin et al., 2020) and can be complementary and alternative medicines for mild COVID-19 patients. For serious and critical COVID-19 cases, some traditional Chinese patent medicines are recommended: 1) for viral infection or mild bacterial co-infection: Xiyanping injection, Duning injection, or Tanreqing injection; 2) for high fever with disturbance of consciousness: Xingnaojing injection; 3) for systemic inflammatory response syndrome or/and multiple organ failure: Xuebijing injection. 4) immunosuppression: Shenmai injection or Shengmai injection (The national commission of the people's republic of China, 2020g; The national commission of the people's republic of China, 2020h).

## Conclusion

To date, there is still no effective drug to cure COVID-19. Moreover, COVID-19 is still a pandemic as of May 2020, and it will probably become a periodic epidemic. The development of anti-COVID-19 drugs is one of the most important tasks to be done. However, the duration of new drug discovery and development usually last years or even decades. The re-purposed use and re-discovery of marketed drugs has become an expressway for anti-COVID-19 drug development.

When COVID-19 broke out in China, clinicians and researchers lacked knowledge of SARS-CoV-2 and COVID-19. At that time, saving lives was the most important thing to be considered. Many un-approved treatments were therefore tested in the clinic based on the experience of SARS and *in vitro* studies. As the evidence grew, many drugs were not proved to be effective treatments for COVID-19. Also, chloroquine lopinavir/ritonavir and other drugs have been reported to have toxic effects. This is why there have been seven editions of the Chinese Guidelines. When COVID-19 broke out in Europe and American in the second wave, clinicians had already gained more knowledge and could avoid unnecessary tests through evidence-based medicine. European and Americans have also developed corresponding guidelines for COVID-19. There is much to be learned from the self-renewal and self-improvement practices of medical staff under the COVID-19 pandemic. The process of updating guidelines is not only helpful to global anti-COVID-19 practice but is also valuable for mankind, as we are still facing the threat of pandemics of undiscovered pathogens in the future.

## Author Contributions

Z-RC drafted and revised the main body of this paper. YZ modified the content of this paper, JL, H-WP, JZ, H-LZ, L-LL, M-FL, and X-HW helped with drafting and editing. J-HW revised and approved the final version.

## Funding

Z-RC received support from Science and Technology Research Project of Department of education of Jiangxi Province (No. GJJ190067).

## Conflict of Interest

The authors declare that the research was conducted in the absence of any commercial or financial relationships that could be construed as a potential conflict of interest.
